# Evolutionary Insights Based on SNP Haplotypes of Red Pericarp, Grain Size and Starch Synthase Genes in Wild and Cultivated Rice

**DOI:** 10.3389/fpls.2017.00972

**Published:** 2017-06-09

**Authors:** Nisha Singh, Balwant Singh, Vandna Rai, Sukhjeet Sidhu, Ashok K. Singh, Nagendra K. Singh

**Affiliations:** ^1^ICAR – National Research Centre on Plant BiotechnologyNew Delhi, India; ^2^Shaheed Udham Singh College of Engineering & Technology, I. K. Gujral Punjab Technical UniversityJalandhar, India; ^3^Divisions of Genetics, ICAR – Indian Agricultural Research InstituteNew Delhi, India

**Keywords:** domestication, haplotype, grain size, red pericarp, starch synthase, wild rice

## Abstract

The origin and domestication of rice has been a subject of considerable debate in the post-genomic era. Rice varieties have been categorized based on isozyme and DNA markers into two broad cultivar groups, Indica and Japonica. Among other well-known cultivar groups Aus varieties are closer to Indica and Aromatic varieties including Basmati are closer to Japonica, while deep-water rice varieties share kinship to both Indica and Japonica cultivar groups. Here, we analyzed haplotype networks and phylogenetic relationships in a diverse set of genotypes including Indian *Oryza nivara/Oryza rufipogon* wild rice accessions and representative varieties of four rice cultivar groups based on pericarp color (*Rc*), grain size (*GS3*) and eight different starch synthase genes (*GBSSI, SSSI, SSIIa, SSIIb, SSIIIa, SSIIIb, SSIVa*, and *SSIVb*). Aus cultivars appear to have the most ancient origin as they shared the maximum number of haplotypes with the wild rice populations, while Indica, Japonica and Aromatic cultivar groups showed varying phylogenetic origins of these genes. Starch synthase genes showed higher variability in cultivated rice than wild rice populations, suggesting diversified selection during and after domestication. *O. nivara/O. rufipogon* wild rice accessions belonging to different sub-populations shared common haplotypes for all the 10 genes analyzed. Our results support polyphyletic origin of cultivated rice with a complex pattern of migration of domestication alleles from wild to different rice cultivar groups. The findings provide novel insights into evolutionary and domestication history of rice and will help utilization of wild rice germplasm for genetic improvement of rice cultivars.

## Introduction

Rice is the most important food crop of Asia, and its domestication began at least 9,000 years ago, although geographical location(s) of its origin has become controversial (Zeder, 2011; Zou et al., 2015). Archeological evidence shows rice domestication beginning 8,000–9,000 years ago in the Yangtze valley (Liu et al., 2007; Fuller et al., 2010) as well as an independent origin of ancestral Indica or proto-Indica rice in the Ganges valley (Fuller, 2011; Prasad et al., 2011). Molecular dating based on synonymous substitution rates suggest that Indica and Japonica rice have evolved from separate wild rice populations that diverged 0.4 to 0.2 million years ago (Vitte et al., 2004; Zhu and Ge, 2005). During the process of domestication and continuous selection for desirable traits, traditional farmers have slowly transformed wild rice into cultivated rice varieties, which is now a pivotal cereal crop for large segment of the human population. Rice has two genetically divergent broad groups of cultivars, namely Indica and Japonica, based on genome wide SSR markers and selected chloroplast genes (Garris et al., 2005). Rice cultivars have been subdivided based on 15 isozyme loci into six genetically distinct sub-populations namely Aus, Indica, Japonica, Aromatic, Rayada and Ashina (Glaszmann, 1987). The Aus cultivars are also known as ‘Aush’ (in Bengal) and ‘Ahu’ (in Assam), meaning ‘early,’ these are sown in summer with the pre-monsoonal showers and harvested in autumn. Aus cultivars are grown primarily in India and Bangladesh during the short growing season under upland conditions. They are known to carry genes for abiotic stress tolerance including drought, heat and salinity. A clear understanding of the evolutionary relationships of rice progenitor species is essential in directing our efforts to search for agronomically useful genes in the wild rice germplasm (Doebley et al., 2006). Two major hypotheses have been proposed on the origin and domestication of rice: (i) single (monophyletic) origin model suggesting that Indica and Japonica cultivars were domesticated at a single location from the wild rice *O. rufipogon* (Molina et al., 2011; Huang et al., 2012). (ii) Independent domestication at more than one location (polyphyletic) model suggesting that the major groups of rice cultivars were domesticated separately in different parts of Asia from separate wild rice ancestors (Vitte et al., 2004; Londo et al., 2006; Civan et al., 2015; Singh et al., 2015). Most recent study on 286 diverse *O. rufipogon* accessions originating from 15 countries using 113,739 nuclear single nucleotide polymorphisms (SNPs) and 25 polymorphic sites in chloroplast sequence has confirmed that three separate wild rice sub-populations were genetically and geographically closely related to Indica, Aus, and Japonica cultivar groups of *O. sativa* (Kim et al., 2016).

Uneven maturity and seed shattering make wild rice unsuitable for cultivation, although human tribes are known to harvest wild rice seeds from natural populations in innovative ways. Rice was domesticated for cultivation making use of one or more of non-shattering mutations in the *SH4* (Zhang et al., 2009), *qSH1* (Konishi et al., 2006) *sh-h* (Ji et al., 2010), and *SHAT1* (Zhou et al., 2012) genes, which subsequently have spread to wider geographic areas. During rice domestication selection pressure would have favored high seed producing populations and increased inbreeding leading to fixation of domestication alleles. Introgressions from wild rice into domesticated rice could readily have occurred enabling domesticated rice to adapt to new environments. Most of the wild rice germplasm in the gene banks have been collected from human-made habitats such as roadside, ditches, and irrigation channels in the areas where such introgression may have occurred. In Asia, repeated introgression from wild rice to cultivated rice is more likely to have occurred in Indica varieties than Japonica varieties because of the locations where these two varietal groups are grown (Sweeney and Mccouch, 2007).

In recent years, several agronomically important rice genes have been cloned and characterized at the molecular level, which has led to an enhanced understanding of rice domestication. An ideal rice cultivar should have high grain yield potential with desirable grain size, quality, nutritional value, disease resistance and abiotic stress tolerance. Some of the important domestication related genes cloned in rice include *SH4* and *qSH1*genes for reduction in grain shattering (Konishi et al., 2006; Li et al., 2006), *Rc* gene for red pericarp color (Sweeney et al., 2006), *Bh4* gene for brown hull color (Vigueira et al., 2013) *GS3* gene for grain size (Fan et al., 2006), *An2* gene for awn length (Gu et al., 2015), *GAD1* gene for grain number, grain length and awn development (Jin et al., 2016), *BADH2* gene for grain fragrance (Bradbury et al., 2005), *CKX1* gene for grain number (Ashikari et al., 2005), *GW2* grain weight (Song et al., 2007) and *GBSS1* gene for amylose content and cooked rice stickiness (Yamanaka et al., 2004). Studies on sequence variation in these genes have aided in tracing the origin and domestication of rice (Civan et al., 2015). In addition to human selection for specific traits, the environment where crops are grown also may have played a role in selection and changes in the genetic diversity of rice (Choudhury et al., 2014). Domestication related genes have experienced severe genetic bottleneck due to selection pressure by tribal farmers and modern plant breeding whereas, enormous genetic diversity exists in their wild ancestors (Doebley et al., 2006; Zhu et al., 2007). Although some alleles exist only within specific rice cultivar groups, as would be expected if multiple domestications occurred independently, other major domestication alleles are claimed to be common to diverse cultivar groups of *O. sativa* (Kovach et al., 2007). Most of the earlier studies have traced the origin and domestication of rice based on genome wide SNPs, but gene based approach offers means of inferring the origin and dispersal of specific traits that have been favored over the course of rice domestication and breeding.

Pericarp color controlled by *Rc* gene located on rice chromosome 7 is an important hallmark trait to examine rice domestication (Gross and Olsen, 2010). The vast majority of rice cultivars have white (non-pigmented) pericarp whereas wild rice species mostly possess dark red pericarp (Sweeney et al., 2006, 2007). The genetic basis of white pericarp in *O. sativa* has been identified as loss of function mutations of the *Rc* gene, a regulatory protein in the proanthocyanidin synthesis pathway. The loss of function mutation in this gene prevents development of a pigmented pericarp layer (Sweeney et al., 2006). Another crucial trait involved in rice domestication is grain size, which in addition to other minor genes is controlled by a major gene *GS3* located on rice chromosome 3. *GS3* was one of the first cloned genes underlying a major QTL controlling grain length and weight. It codes for a protein with several conserved domains including a phosphatidylethanolamine-binding protein (PEBP)-like domain and a *trans*-membrane region and mutations in this gene leading to both increased and decreased grain length as compared to the wild rice have been reported in the cultivated rice (Fan et al., 2006; Anand et al., 2015). Grain starch quality is another important trait in cereals that has been the target of selection during domestication due to its direct impact on cooking and eating quality (Whitt et al., 2002). Starch, accounts for more than 90% of the dry milled rice grains and is a major determinant of both rice yield and quality (Mohapatra et al., 1993). The *waxy* locus functionally known as *GBSSI* gene is one of the extensively studied genes of rice and is highly associated with the taste and texture of cooked rice. This locus provides much information regarding how an important mutation spreads in a crop gene pool. It also provides information related to the impact of human selection particularly on rice quality. Starch synthesis pathway is well characterized in plants and until now more than 20 genes involved in the starch synthesis have been identified in the cereal crops. Among these, six genes are known to play major role in rice endosperm starch synthesis: Shrunken2 (*Sh2*), Brittle2 (*Bt2*), Waxy (*Wx*), Starch synthase IIa (*SSIIa*), Starch branching enzyme IIb (*SbeIIb*), and Isoamylase1 (*Iso1*) (James et al., 2003). *GBSSI* and *SSIIa* are major genes involved in important grain quality attributes of rice such as amylose content and gelatinization temperature. Starch synthase genes *SSI, SSII, SSIII*, and *SSIV* are responsible for amylopectin chain elongation and their distribution between the granular and soluble fractions (Ball and Morell, 2003; Li et al., 2003). The origin of starch synthase subfamilies is clearly ancient and will provide important clues to rice domestication, given the conservation of their orthologs from *Chlamydomonas* through to the dicots and monocots (Ball and Morell, 2003).

In the present study we investigated the SNP haplotype variation in *Rc* gene for pericarp color, *GS3* gene for grain size and eight different starch synthase genes in a large set of wild rice accessions together with representative varieties of major rice cultivar groups to understand the process of rice evolution and domestication by employing haplotype network and phylogenetic analysis.

## Materials and Methods

### Plant Materials

A set of 202 diverse genotypes was used for the analysis of gene haplotype network and phylogenetic relationships among Indian *Oryza nivara/Oryza rufipogon* wild rice accessions and different rice cultivar groups. The set included a 182 wild rice accessions collected from different geographical regions of India; 91 from Mid-Gangetic Plains (MGP), 23 from West-Himalayan Region (WHR), 18 from West Coastal Plains (WCP), 17 from East Plateau Hills (EPH), 9 from Gujarat Plains and Hills (GPH), 7 from Lower Gangetic Plains (LGP), 6 from East Coastal Plains (ECP), 6 from East Himalayan Region (EHR), and 5 from Upper Gangetic Plains (UGP). These wild rice accessions represented admixture-free genotypes representing three different pure model based sub-populations (Pro-Indica, Pro-Aus, and Mid-Gangetic) of Indian *O. nivara*/*O. rufipogon* wild rice accessions (Fst values ≥ 0.9, our unpublished results). In addition five of each genotype of Indica, Japonica, Aromatic, and Aus cultivar groups were also included in the analysis. Details of the wild rice accessions and rice cultivars used in present study are provided in Supplementary Table S1 and our online wild rice database^1^.

### DNA Extraction and SNP Array Hybridisation

Genomic DNA was extracted from young green leaves using the method of Murray and Thompson, 1980 with minor modifications, quantified using Nano-drop spectrophotometer absorbance at 260/280 nm and quality checked by electrophoresis in 1% agarose gel. For SNP genotyping an Affymetrix 50K genic SNP chip ‘OsSNPnks’ described earlier was used (Singh et al., 2015). For target probe preparation, 20 μL of genomic DNA was used for each sample, with DNA concentration of 10 ng/μL (200 ng DNA in 20 μL) according to Affymetrix Axiom^®^ 2.0 Assay Manual. DNA amplification, fragmentation, chip hybridisation, single-base extension through DNA ligation and signal amplification were performed using Affymetrix Axiom^®^ 2.0 Assay Manual Target Prep Protocol QRC (P/N 702990). Staining and scanning were performed on the GeneTitan^®^ Multi-Channel instrument according to manufacturer’s instructions ^2^.

### SNP Calling and Data Analysis

SNP genotypes were called using Affymetrix Genotyping Console^TM^v4.1 and Axiom^TM^ Suite. SNPs with low call rates across all samples were removed from the dataset, and high-performing SNPs with a DQC of >0.85 and call rates of >95.0% were used for analyses. To evaluate the data quality the dataset was imported in other compatible formats, such as PLINK (Purcell et al., 2007) and text, was examined using two different softwares, APT with R package and SNPolisher^3^. Out of the large number of SNPs on the chip we extracted SNPs in 10 agronomically important domestication related rice genes for haplotype and phylogenetic analysis (**Table 1**).

**Table 1:** Details of genes with number of SNPs, haplotypes and number of rice accessions used for haplotype network and phylogenetic analysis.

S.L. No.	Gene Name	LOC/Gene Id.	Total number of SNPs	Number of haplotypes	Number of genotypes
(1)	*Rc*	Os07g11020	4	4	179
(2)	*GS3*	Os03g44500	9	9	180
(3)	*GBSSI*	Os06g0133000	7	8	202
(4)	*SSS1*	Os06g06560	46	17	66
(5)	*SSIIa*	Os06g12450	5	7	175
(6)	*SSIIb*	Os02g51070	4	6	188
(7)	*SSIIIa*	Os08g09230	20	18	84
(8)	*SSIIIb*	Os04g53310	7	11	192
(9)	*SSIVa*	Os01g52250	19	12	110
(10)	*SSIVb*	Os05g45720	9	9	150

### Haplotype Network and Phylogenetic Tree Construction

SNP haplotypes for the selected genes were generated using TASSEL 3.2.1 (Bradbury et al., 2007). Based on the SNP haplotype analysis for each gene, a phylogenetic tree was constructed using an improved version of the neighbor-joining algorithm, and visualized using FigTree v1.4.0 (Rambaut, 2009). Haplotype network for each gene was constructed for analysis of genealogical relationship among the haplotypes using Network software^4^ (Bandelt et al., 1999) and haplotype diversity was calculated with the DnaSP software version 5.10^5^ (Rozas et al., 2003).

## Results and Discussion

### *Rc* Gene for Red Pericarp

**FIGURE 1:**
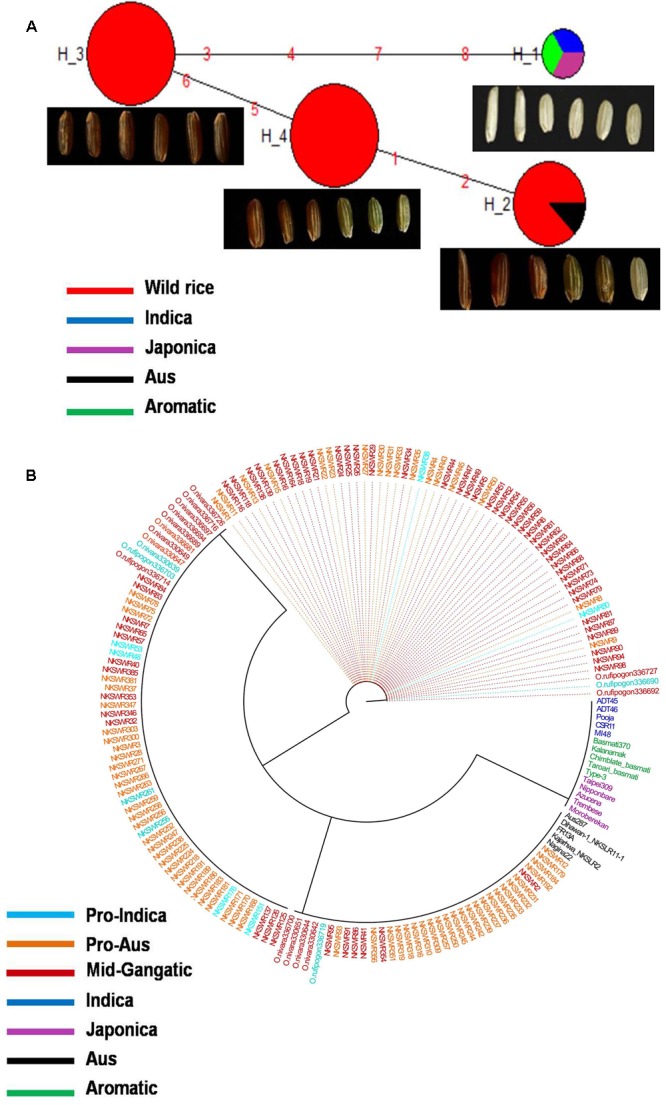
**(A)** A haplotype network of *Rc* gene along with its pericarp color, in total four haplotypes were formed, *Rc*-H3 was the ancestral haplotype of wild rice. The size of each circle is proportional to haplotype (allele) frequency. To determine the origin and dispersal of these mutations in *Rc* gene we examined in total 179 diverse rice genotypes. Color-coding represents different population groups (Red-Wild rice, Blue-Indica, Black-Aus, Green-Aromatic, and Magenta-Japonica). **(B)** Haplotype based phylogenetic tree of *Rc* gene was constructed using 179 diverse rice genotypes and were separated into four major distinct groups. Color-coding represents different varietal groups (Cyan-Pro-Indica, Orange-Pro-Aus, Red-Mid-Gangetic, Blue-Indica, Black-Aus, Green-Aromatic, and Magenta-Japonica).

Haplotype variation in *Rc* gene was examined in 179 geographically and genetically diverse genotypes, including 159 wild rice accessions and five each of representative varieties from Aus, Indica, Japonica, and Aromatic rice cultivar groups. Even though additional SNP and SSR have been reported in the *Rc* gene (Sweeney et al., 2006, 2007), present analysis was based on four SNPs in the *Rc* gene that could be genotyped with high quality score using the 50K rice SNP chip (Singh et al., 2015). Haplotype network analysis revealed four haplotypes (*Rc*-H1, *Rc*-H2, *Rc*-H3, and *Rc*-H4) with a haplotype diversity (Hd) of 0.7076. *Rc*-H3 was the most ancestral haplotype located at the root of the NJ phylogenetic tree (**Figures 1A,B**). Interestingly, *Rc*-H3 haplotype was present in 62 wild rice accessions all belonging to the MGP region, but it was represented by all three structure based sub-populations of wild rice, *viz*. ‘Pro-Indica,’ ‘Pro-Aus,’ and ‘Mid-Gangetic types, although predominantly in the Mid-Gangetic sub-population. All wild rice accessions with *Rc*-H3 haplotype have red pericarp. The other three haplotypes *Rc*-H1, *Rc*-H2, and *Rc*-H4 were derived from *Rc*-H3 but their origin followed two independent routes, one from *Rc*-H3 to *Rc*-H1 while another one was from *Rc*-H3 to *Rc*-H4 to *Rc*-H2 (**Figure 1A**). Based on NJ phylogenetic tree *Rc-*H1 was the most recently evolved haplotype, which is shared by Indica, Japonica, and Aromatic rice cultivars, all having white pericarp. In contrast, all the Aus cultivars possessed haplotype *Rc*-H2 that was shared by 35 wild rice accessions predominantly from the ‘Pro-Aus’ wild rice sub-population. These ‘Pro-Aus’ accessions were collected from diverse geographical regions of India (Supplementary Table S1). Genotypes with *Rc*-H2 haplotype showed red, green, and white pericarp, suggesting that there are other genes involved in the pericarp color development or there may be further differentiation of the *Rc*-H2 haplotype based on additional SNP information (Cui et al., 2016). While wild rice accessions with this haplotype showed red or green pericarp, the Aus cultivars showed green or white pericarp (**Figures 1A,B**). In any case presence of a different *Rc* allele for white pericarp in Aus cultivars showed independent domestication of Aus cultivars (Civan et al., 2015; Singh et al., 2015). Earlier, a monophyletic origin of cultivated rice has been claimed based on occurrence of common haplotype of *Rc* gene in Japonica and Indica rice cultivar groups.

Haplotype analysis of *Rc* gene conducted earlier by Sweeney et al. (2007) showed that an identical 14 bp deletion was present in 97.9% of the cultivated rice accessions with white pericarp, this deletion was not found in any wild or cultivated rice with red pericarp (Furukawa et al., 2007; Cui et al., 2016). In congruence with our results these studies have also observed, though not highlighted, a second mutation in the *Rc* gene (*Rc-S* allele) for white pericarp in the Aus group of rice cultivars. The 14 bp deletion most likely originated in the Japonica-Aromatic cultivar group and then it crossed both geographic and sterility barriers to move into the Indica cultivars perhaps by cultural exchange among ancient farmers over the course of rice domestication with strong positive selection for white pericarp in both Indica and Japonica cultivar groups. The ancestral haplotype *Rc*-H3 with red color pericarp was present in wild rice accessions along with *Rc*-H2 and *Rc*-H4 haplotypes. The recessive *rc* allele (*Rc*-H1 haplotype in present study) leading to white pericarp is predominant in the modern rice varieties, but our study confirms that white pericarp selected in the independent domestication of Aus cultivars is due to a different mutation in the *Rc* gene, a fact not adequately emphasized in the earlier studies. It showed that different mutations leading to same preferred phenotypic trait have arisen in different wild rice sub-populations leading to separate domestication events. Red pericarp wild rice and landraces carrying functional *Rc* haplotypes are still grown in different parts of India. Wild rice haplotype *Rc*-H3 was the most ancestral haplotype as most of the red pericarp wild rice clustered within this group (**Figures 1A,B**). Selection for non-pigmented grains in rice may potentially reflect human selection against seed dormancy, selection for improved taste and cooking qualities and also with the pleasant white appearance. However, the pigment serves as a powerful antioxidant that has been demonstrated to reduce cardiovascular disease, diabetes and help in cancer prevention (Zhang et al., 2010). In another domestication related rice gene *BADH2*, multiple mutations have been found for the loss of function of gene responsible for grain aroma (Shi et al., 2008). Our results indicate that white pericarp in modern cultivated rice has come from at least two different domestication events in wild rice.

### *GS3* Gene for Grain Length

**FIGURE 2:**
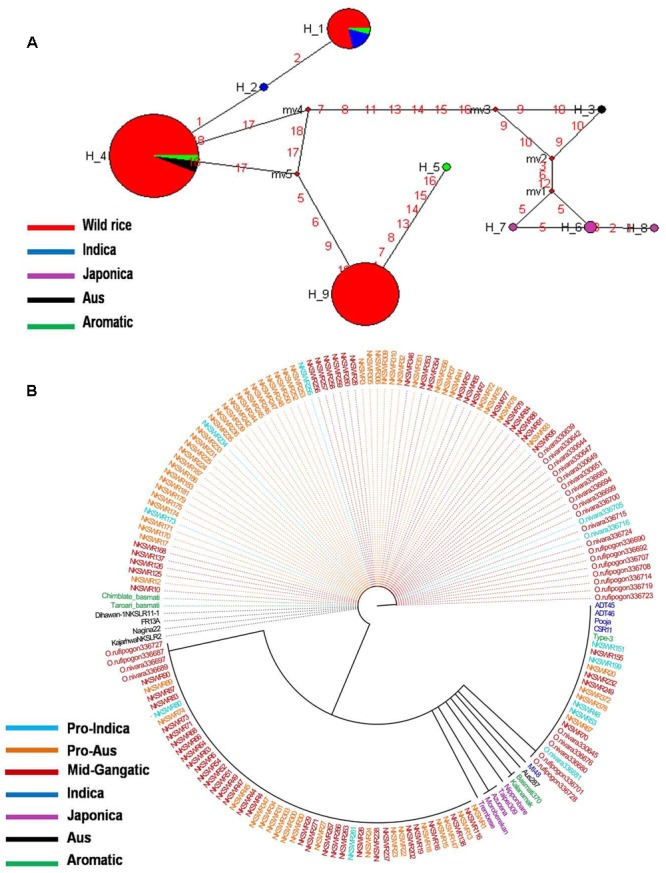
**(A)** Haplotype networks of *GS3* gene, in total nine haplotypes were generated, *GS3-*H4 was the ancestral haplotype representing wild rice along with Aus and Aromatic cultivars. **(B)** Haplotype based phylogenetic tree of *GS3* gene was constructed using 180 diverse rice genotypes and were separated into nine distinct groups. Color-coding represents different sub-populations or varietal groups (Cyan-Pro-Indica, Orange-Pro-Aus, Red-Mid-Gangetic, Blue-Indica, Black-Aus, Green-Aromatic, and Magenta-Japonica).

*GS3* gene located on chromosome 3 is a major gene for grain size in rice, which has also been under strong selection pressure during domestication. Long grain rice varieties are favored in many countries due to their beautiful appearance and high volume expansion upon cooking. There is great diversity of seed size and shape both within and between different sub-populations of rice cultivars, although each sub-population is associated with characteristic seed morphology. Domestication process has resulted in both extra long and extra short rice grains as compared to the wild rice because of different mutations in the *GS3* gene (Zhao et al., 2010; Anand et al., 2015). Here we examined the haplotype network and phylogenetic relationship of *GS3* alleles to find evidence for the origin and selection of domestication alleles and their association with different sub-populations of wild and cultivated of rice. The SNP variation in *GS3* gene was analyzed in 160 diverse wild rice and 20 representative rice cultivars. The haplotype network and phylogenetic tree were constructed based on nine high quality SNPs in the *GS3* gene that identified total nine haplotypes with an ‘Hd’ value of 0.6214. There were three major (*GS3*-H1, *GS3*-H4, and *GS3*-H9) and six minor (*GS3*-H2, *GS3*-H3, *GS3*-H5, *GS3*-H6, *GS3*-H7, and *GS3*-H8) haplotypes. It was clear from the phylogenetic analysis that domestication of *GS3* gene has followed three independent paths originating from the most ancestral wild rice haplotype *GS3*-H4 which was shared by the majority 88 wild rice accessions along with four Aus and four Aromatic rice cultivars (**Figures 2A,B**). The *GS3*-H4 haplotype was shared by all three wild rice sub-populations, namely ‘Pro-Indica,’ ‘Pro-Aus,’ and ‘Mid-Gangetic’ types indicating its ancient origin. Six minor haplotypes were restricted to cultivated rice, indicating their recent origin under human selection. Two of the three major wild rice haplotypes were also shared by the rice cultivars suggesting that a range of seed sizes have been selected by humans during domestication and breeding while wild environment favored optimum size for efficient seed dispersal and survival. Here, *GS3*-H1 was the most versatile haplotype comprising of 4 Indica, 2 Aromatic, and 18 wild rice accessions (eight from MGP, four each from LGP and EPH, and two from WCP regions). The third major haplotype *GS3*-H9 was restricted to 54 wild rice accessions (40 from MGP and 14 from WCP). Five Japonica cultivars possessed three minor haplotyes (*GS3*-H6, *GS3*-H7, and *GS3*-H8), the other three minor haplotypes were represented one each by Indica (*GS*3H2), Aus (*GS3*-H3), and Aromatic (*GS3*-H5) cultivars.

We also calculated the average grain length (GL) and grain width (GW) for the three major haplotypes. The average GL for haplotype *GS3*-H4 was 3.71 mm, average GW 1.39 mm and GL/GW ratio of 2.66 mm. The *GS3*-H9 haplotype comprising entirely of 54 wild rice accessions mostly from the MGP region has longer average GL, GW, and GL/GW ratio of 4.12, 1.44, and 2.86 mm, respectively. The *GS3*-H1 haplotype showed the highest average GL of 3.97 mm, GW of 1.33 mm, and GL/GW of 2.98 mm comprising long slender grains. It shows that short grain Aus and Aromatic rice cultivars were domesticated first then subsequent selective breeding by generations of farmers, diversified it into several different grain sizes based on selection of random mutants and recombination with other genes in the domesticated rice, for improved yield, quality and other agronomic or cultural value traits (Deb, 2005). Five Japonica cultivars known for their short, round shape and sticky cooked grains showed most diverse haplotypes derived from mutations in the ancestral wild rice haplotype. Similarly, Indica and Aus cultivars are known for long slender grains while Aromatic rice varieties have both long and short grains, which seem to be directly associated with their *GS3* haplotype. In a previous study on the haplotype analysis of 54 diverse rice accessions of 86 SNPs and 28 indels were identified in a 6.57 kb *GS3* DNA, suggesting origin of long grain allele in a Japonica type ancestor and subsequent introgression into the Indica gene pool (Takano-Kai et al., 2009). But *GS3* haplotype network and phylogenetic analysis here shows independent path of the evolution of Japonica and Indica haplotypes, while major Aus, Indica, and Aromatic rice haplotypes were shared with the wild rice accessions. Earlier, haplotype analysis of 282 diverse rice cultivars has also indicated multiple independent origins of short seeded *GS3* alleles suggesting that farmers and early breeders forced artificial selection favoring short seeds (Takano-Kai et al., 2013; Anand et al., 2015). Intercrossing of short and long grain varieties has resulted into evolution of intermediated grain size varieties and modern superior varieties are the result of domestication and artificial selection on *GS3* and other grain size genes, including *qGL7.1* and *qGL1.1* (Amarawathi et al., 2008; Bai et al., 2010; Anand et al., 2015). A *GS3* haplotype conferring extra short grain was selected by traditional Indian farmers (Anand et al., 2015).

The preference for grain size and shape varies from one group of consumers to another. Generally long slender grains are preferred in Southeast Asia while Chinese consumers prefer short and bold rice grains. Indian consumers like rice with diverse grain size ranging from very short to extra-long grains with shapes ranging from bold to slender. Consumers in Indonesia and Bangladesh prefer medium slender grains. Japanese, Taiwanese, and South Koreans consume short and bold rice grains, whereas in several states of India, and Sri Lanka, both short and medium grain types are popular (Calingacion et al., 2014). All the short and medium grains genotypes carry the C-allele and the long and extra-long grains carry the A-allele of the *GS3* gene (Fan et al., 2009). Further, the grain size and shape have direct impact on yield, head rice recovery during milling. Although popular Basmati rice fetching premium price in the international market has long slender grains, other indigenous Indian aromatic rice varieties have short or medium seed size.

### *GBSSI* (*waxy*) Gene for Amylose Starch Synthesis

Starch is a major component of the human diet and constitutes about 90% of the total dry weight of polished rice. It occurs in two forms: amylose (15–30%), consisting of predominantly linear chains of glucose monomers linked by α 1–4 glycosidic bonds, and amylopectin (70–85%), in which the chains are branched by the addition of α1–6 glycosidic bonds. Starch property has been under strong selection pressure in rice domestication as it directly affects the cooking and eating quality of the rice. Rice genome has five sub-families of starch synthases, namely granule bound starch synthase (GBSS) and soluble starch synthase I, II, III, and IV (*SSI, SSII, SSIII*, and *SSIV*). Each class of soluble starch synthase plays a distinct role in the synthesis of amylopectin. *GBSSI* also kwon as *Waxy* (*Wx*) gene located on rice chromosome 6 encodes a granule-bound starch synthase that is required for amylose synthesis in rice grains has played a crucial role in rice domestication (Hirano et al., 1998; Isshiki et al., 1998). However, other minor genes such as *dull* and *amylose extender* (*ae*) are also reported to play important roles in modification of amylose in rice (Isshiki et al., 2000). The *Wx* genes have been cloned in *O. sativa* (Wang et al., 1990; Hirano and Sano, 1991; Okagaki, 1992) and *O. glaberrima* (Umeda et al., 1991). Two wild-type alleles, *Wxa* and *Wxb*, were found at the *waxy* locus in cultivated rice. *Wxa* is characteristic of Indica rice, while *Wxb* is found in Japonica rice (Sano, 1984). Mutation in this gene drastically reduced the amylose synthesis in glutinous rice varieties. Glutinous rice contains a G to T mutation at the 59 splice site of *Wx* intron 1, which leads to incomplete post-transcriptional processing of the pre-mRNA (Wang et al., 1995; Frances et al., 1998; Hirano et al., 1998; Isshiki et al., 1998). Rice cultivars are categorized based on amylose content as waxy (0–5%), very low (5–12%), low (12–20%), intermediate (20–25%), and high (25–33%). Wild rice does not have glutinous starch which was selected only after domestication, and evolution of glutinous rice may have occurred in various stages (Vaughan et al., 2008). Japonica rice varieties having low amylose content (2–8%) are sticky after cooking as compared to long-grained Indica and Aus varieties. Expression of *GBSSI* gene is developmentally regulated as it is highly expressed during seed development, which is crucial for amylose synthesis (Sano, 1984; Hirose and Terao, 2004; Ohdan et al., 2005; Fujita et al., 2011). The *waxy* mutation creating a SNP in exon four of the *GBSSI* gene reduces the binding of *GBSSI* enzyme to starch granules resulting in low amylose synthesis (Liu et al., 2009).

**FIGURE 3:**
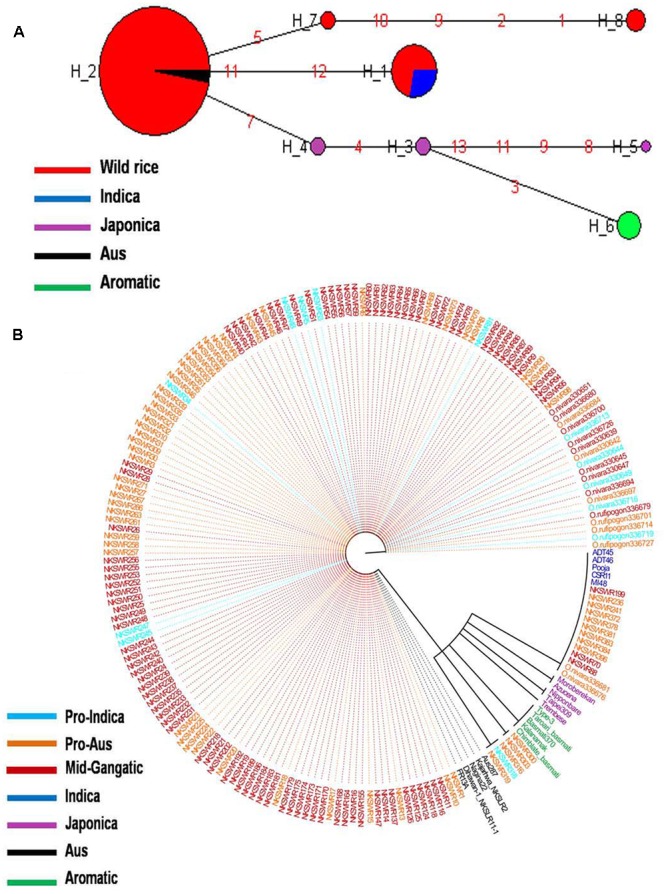
**(A)** Haplotype networks of *GBSSI* gene, in total eight haplotypes were formed, *GBSS1*-H2 was the major ancestral haplotype of wild rice sub-populations, shared with Aus cultivars. **(B)** Haplotype based phylogenetic tree of *GBSSI* gene was constructed using 202 diverse rice genotypes and were separated into eight distinct groups. Color coding represents different varietal groups (Cyan-Pro-Indica, Orange-Pro-Aus, Red-Mid-Gangetic, Blue-Indica, Black-Aus, Green-Aromatic, and Magenta-Japonica).

Haplotype network and phylogenetic analysis of *GBSSI* gene was performed using 8 SNPs in a set of 182 wild rice and 20 diverse rice cultivars resulting in eight haplotypes with low ‘Hd’ value of 0.2994. There were two major haplotypes designated *GBSSI*-H1 and *GBSSI*-H2 and six minor haplotypes designated *GBSSI*-H3 to *GBSSI*-H8. Similar to that with the *GS3*, wild rice accessions showed significantly less haplotype diversity than rice cultivars. *GBSSI*-H2 was by far the most predominant and also most ancient haplotype from which the remaining seven haplotypes were derived. It was highly conserved in 164 of the 182 wild rice accessions collected from diverse geographical regions of India (97 MGP, 26 EPH, 14 WHR, 12 UGP, 10 LGP, and 5 WCP) and was also shared by five Aus cultivars, once again showing that Aus cultivars have the most ancient of the four rice cultivar groups. All three sub-populations of wild rice, ‘Pro-Indica,’ ‘Pro-Aus,’ and ‘Mid-Gangetic’ were represented by the ancestral *GBSSI*-H4 haplotype. Evolution of *GBSS1* gene in rice cultivars has taken three independent paths with Aus cultivars having the most ancient *GBSS1*-H2 haplotype, which was also conserved in 89.1% of the wild rice accessions. The *GBSSI*-H1 haplotype represented 13 wild rice accessions from wide geographical regions (6 EHR, 3 MGP, 2 each from EPH and WCP) together with all five Indica varieties. Of the 13 wild rice accessions sharing *GBSSI*-H1 haplotype with the Indica rice cultivars, nine were from ‘Pro-Aus’ and three from ‘Mid-Gangetic’ sub-populations, but none were from Pro-Indica group, suggesting that the *GBSS1* haplotype in the Indica rice cultivars has its origin in non-Pro-Indica wild rice ancestors. The five Indica rice cultivars used here possess superior eating quality which is also reported for several wild rice populations harvested by the tribal in different parts of India. Three minor haplotypes *GBSSI*-H3, *GBSSI*-H4, and *GBSSI*-H5 of Japonica varieties were derived from the ancestral haplotype *GBSSI*-H2. Interestingly, all the five aromatic rice cultivars both short grain and long grain basmati type share a common haplotype *GBSSI*-H6, which was derived from Japonica haplotype *GBSSI*-H3. Two more minor haplotypes *GBSSI*-H7 and *GBSSI*-H8 were found uniquely in the wild rice accessions collected from the WCP region (**Figures 3A,B**).

It was exciting to see that similar to the grain size gene *GS3, GBSSI* gene was also more conserved in wild rice than in cultivated rice. While 182 wild rice genotypes showed only four haplotypes, only 20 rice cultivars possessed six different haplotypes two of which were shared by wild rice. The highly conserved haplotype *GBSS1*-H2 present in 89.1% of the wild rice accessions must have selective advantage for survival in natural environment. On the other hand large number of haplotypes have been selected during the domestication and breeding of rice cultivars for various user preferences of cooking and eating quality. These results also showed three independent origins of cultivated rice, the most ancestral haplotype is shared by the Aus cultivars which have intermediate amylose content, the second haplotype is common to high amylose Indica cultivars that is shared by another small group of mostly Pro-Aus wild rice accessions and the third clade is that of Japonica and Aromatic rice cultivars which have low to intermediate amylose content. Japonica cultivars showed the maximum number of three haplotypes in just five varieties and Aromatic cultivars including both short grain and basmati rice have single haplotype originating from one of the Japonica haplotypes. These results are consistent with recent genome wide phylogenetic studies showing tri-phyletic origin of domesticated rice (Civan et al., 2015; Singh et al., 2015; Kim et al., 2016).

The three main properties that determine rice eating and cooking quality are, amylose content, gel consistency, and gelatinization temperature, which also correlate with one another. Grain amylose content is the major determinant of rice eating and cooking qualities. High amylose rice is mainly found in tropical countries while low amylose rice is preferred in temperate countries. High amylose rice varieties are less sticky, have low glycemic index which is beneficial for diabetics. Rice with intermediate amylose content is preferred in Iran, Laos, Pakistan, Malaysia, Philippines, India, and some provinces of China, Vietnam, Indonesia, and Uruguay. High amylose and low amounts of amylopectin varieties are popular in Myanmar, Sri Lanka, provinces of Indonesia, and many states of India (Calingacion et al., 2014).

### *SSSI* Gene for Amylopectin Synthesis

**FIGURE 4:**
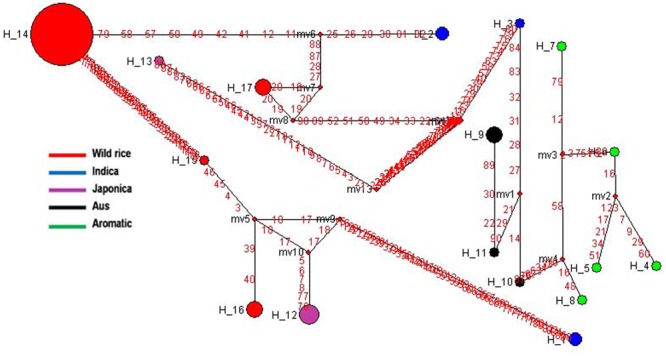
Haplotype networks of *SSSI* gene, in total 17 haplotypes were generated, and *SSS1*-H14 was the ancestral haplotype.

Soluble Starch Synthase I (*SSSI* gene) located very close to the *GBSS1* gene on rice chromosome 6 is responsible for the formation of α 1–4 glycosidic bonds in amylopectin. The expression of *SSSI* gene in Indica rice cultivars is lower than that of Japonica rice which reduces the synthesis of short chains in Indica rice amylopectin (Takemoto-Kuno et al., 2006). Haplotype network and phylogenetic analysis of *SSSI* gene was performed based on 46 SNPs in a set of 66 diverse rice genotypes including 46 wild rice accessions and 20 representative rice cultivars. Total 17 haplotypes were generated, one major (*SSSI*-H14), the remaining 16 were minor haplotypes with overall ‘Hd’ value of 0.6466. *SSSI*-H14 was the most ancestral haplotype present in 39 wild rice accessions, all from the MGP region. Indica and Aus cultivars were represented by three haplotypes each, *SSSI*-H1-*SSSI*-H3 and *SSSI*-H9-*SSSI*-H11, respectively. Five Japonica cultivars were represented by two minor haplotypes (*SSSI*-H12 and *SSSI*-H13). Each of the five aromatic cultivars possessed a different haplotype (*SSSI*-H4-*SSSI*-H8). There were also three minor haplotypes (*SSSI*-H15-*SSSI*-H17) of wild rice derived from *SSSI*-H14 (**Figure 4** and **Supplementary Figure S1**). The results showed that Japonica, Indica, Aus, and Aromatic rice cultivars have evolved from separate ancestral wild rice groups and then further separated into minor haplo-groups, supporting polyphyletic origin of rice.

### *SSIIa* and *SSIIb* Genes for Amylopectin Synthesis

**FIGURE 5:**
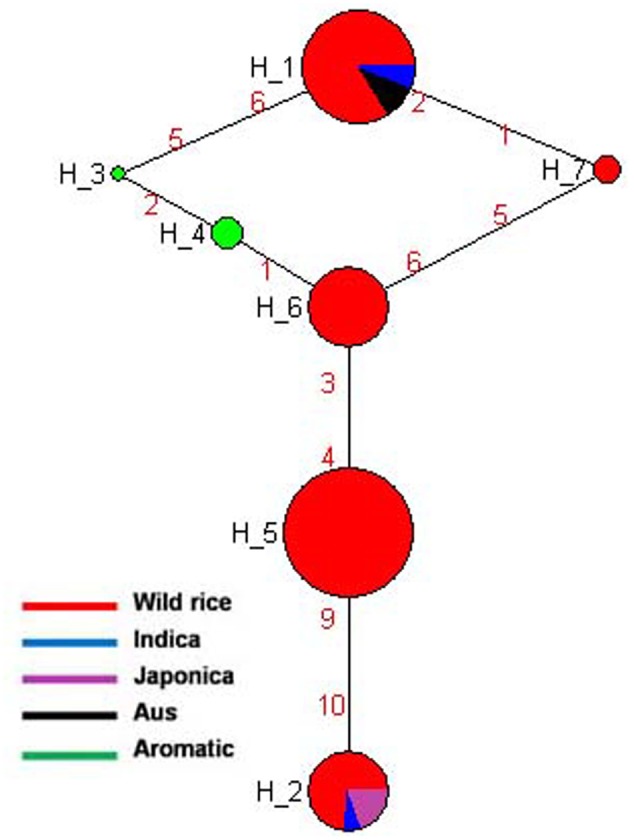
Haplotype networks of *SSIIa* gene, in total seven haplotypes were generated, and *SSIIa*-H5 was the ancestral haplotype.

*SSIIa* is known to have a major affect on starch quality and located at the *alk* locus on the short arm of chromosome 6 in the rice genome (Umemoto et al., 2002). This gene is predominantly expressed in the endosperm at very high level and presumably affects amylopectin structure (Morell et al., 2003). The effect of this gene on cooking quality and starch texture has clearly been revealed (Umemoto et al., 2004, 2008). Gelatinization temperature of rice starch is mainly determined by *SSIIa* gene (Luo et al., 2015). Previously genotyped 60 rice cultivars for two SNPs of *SSIIa* gene and also incorporated three other SNPs of this gene generated five haplotyes. These SNPs alter the branch-length distribution of amylopectin and gelatinization properties and alkali spreading value of rice (Umemoto and Aoki, 2005). In the present study, total five SNPs of *SSIIa* gene were genotyped for the construction of haplotype network and phylogenetic tree of 175 diverse genotypes including 155 wild rice and 20 rice cultivars. Total seven haplotypes were generated of which four were major (*SSIIa*-H1, *SSIIa*-H2, *SSIIa*-H5, and *SSIIa*-H6) and three were minor (*SSIIa*-H3, *SSIIa*-H4, and *SSIIa*-H7) with ‘Hd’ value of 0.7387. The *SSIIa*-H5 haplotype present in the wild rice accessions collected from MGP and UGP regions was the ancestral haplotype for the other six haplotypes. However, *SSIIa*-H2 and *SSIIa*-H6 were closest to *SSIIa*-H5 with their allele flow in two opposite directions. *SSIIa*-H2 haplotype was shared by all three groups of wild rice, all five Japonica cultivars and two Indica cultivars, while *SSIIa*-H6 was represented by wild rice only. *SSIIa*-H1 was shared by wild rice, all of the Aus and most of the Indica cultivars (**Figure 5** and **Supplementary Figure S2**). The aromatic rice varieties showed two minor haplotypes, *SSIIa*-H3 and *SSIIa*-H4, whereas another minor haplotype *SSIIa*-H7 was present in three wild rice accessions, collected 1 from MGP and 2 from EPH regions. The phylogenetic analysis of *SSIIa* gene also showed a polyphyletic origin of cultivated rice because rice cultivar groups have followed three independent evolutionary pathways. Recently, nucleotide diversity and molecular evolution based on 93 SNPs in this gene was analyzed in 199 Chinese wild rice accessions and 122 cultivated accessions, also reporting three different haplotypes in both wild and cultivated rice accessions. Their hypothesis was also supported that cultivated rice was independently domesticated from multiple domestication and has undergone balancing selection on separate haplotypes in multiple populations in China (Zhou et al., 2016).

**FIGURE 6:**
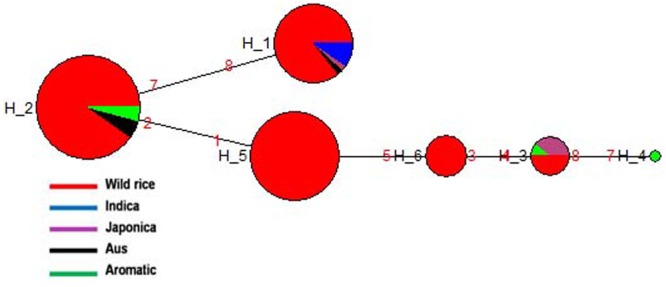
Haplotype networks of *SSIIb* gene six haplotypes were generated, *SSIIb*-H2 was the ancestral haplotype comprising large number of wild rice genotypes, shared with Aus and Aromatic cultivar groups.

*SSIIb* gene located on rice chromosome 2 is expressed primarily in leaf blades and sheaths (leaf specific) at early stage of grain filling (Hirose and Terao, 2004). A total of 188 diverse rice genotypes were analyzed based on four SNPs in the *SSIIb* gene. Both haplotype network and phylogenetic analysis showed similar pattern of gene diversification. In total six haplotypes were generated, three major (*SSIIb*-H1, *SSIIb*-H2, and *SSIIb*-H5) and three minor (*SSIIb*-H3, *SSIIb*-H4, and *SSIIb*-H6) haplotypes with ‘Hd’-0.7256. *SSIIb*-H2 was the ancestral haplotype shared by majority of the wild rice accessions from diverse geographical regions (EHR, MGP, UGP, and WCP) along with four Aus and three Aromatic cultivars. The other five haplotypes were derived from this ancestral haplotype. Here also we can see a tri-phyletic origin of the *SSIIb* haplotypes in rice cultivars the ancestral *SSIIb*-H2 was shared by Aus and Aromatic cultivars, *SSIIb*-H1 was present in all Indica rice cultivars, while in another evolutionary route, comprising of 4 different haplotypes (*SSIIb-*H3 to *SSIIb*-H6), two haplotypes (*SSIIb*-H5 and *SSIIb*-H6) were solely represented by wild rice genotypes. Wild rice accessions belong to MGP and UGP regions were clustered in *SSIIb*-H5 group while *SSIIb*-H6 haplotype was specific to the GPH region. Japonica cultivars showed two haplotypes (*SSIIb*-H1 and *SSIIb*-H3) but *SSIIb*-H3 was the major haplotype present in wild rice, four Japonica and one Aromatic cultivars (**Figure 6** and **Supplementary Figure S3**). These results showed that Indica and Japonica cultivars groups have originated from wild rice genotypes through independent phylogenetic routes while haplotype of Aus cultivars was the most ancient and still shared by diverse wild rice genotypes.

### *SSIIIa* and *SSIIIb* Genes for Amylopectin Synthesis

**FIGURE 7:**
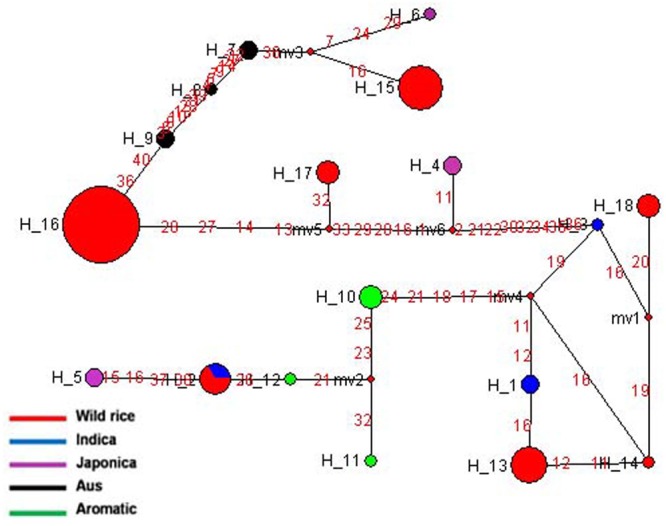
Haplotype networks of *SSIIIa* gene, in total 18 haplotypes were generated, and *SSIIIa*-H16 was the ancestral haplotype of wild rice.

The *SSIIIa* gene located on chromosome 8 is highly expressed in endosperm and green tissues. Haplotype network and phylogenetic analysis was done based on 20 SNPs of *SSIIIa* gene in 84 diverse rice genotypes, which was nicely classified into 18 distinct haplotypes with a haplotype diversity ‘Hd’ value of 0.8153. Of these *SSIIIa*-H15 and *SSIIIa*-H16 were the major haplotypes present entirely in the wild rice accessions. Here, *SSIIIa*-H16 was the most ancestral haplotype comprising of wild rice accessions from EHR, UGP, WCP, and EPH region. Five haplotypes of wild rice (*SSIIIa*-H13-*SSIIIa*-H15, *SSIIIa*-H17- and *SSIIIa*-H18) belonged to different geographical regions of India (**Figure 7** and **Supplementary Figure S4**). Three haplotypes were formed in Aromatic, Aus, and Indica. Similarly, Japonica cultivars also carried three minor haplotypes, *SSIIIa*-H4*-SSIIIa*-H6. Here also the cultivated rice showed more variations than wild rice, suggesting selection for diverse end-usage. Aus and Indica cultivars were derived from the same ancestral wild rice but grouped into separate haplotypes. Hence, this gene also supports the hypothesis of polyphyletic origin of cultivated rice, because all the four cultivar groups show multiple independent origins.

**FIGURE 8:**
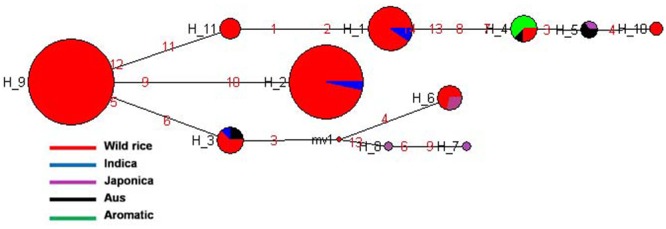
Haplotype networks of *SSIIIb* gene, 11 haplotypes were generated, and *SSIIIb*-H9 was the ancestral haplotype of wild rice.

*SSIIIb* gene located on rice chromosome 4 is expressed mainly in endosperm but also in leaf sheaths. It showed 11 haplotypes based on 7 SNPs in 192 diverse rice genotypes. *SSIIIb*-H2 and *SSIIIb*-H9 were the two major haplotypes along with nine minor haplotypes with ‘Hd’ value of 0.7275. *SSIIIb*-H9 was the most predominant ancestral haplotype present in 77 wild rice accessions and was the source of origin for remaining 10 haplotypes. The ancestral wild rice haplotype was prevalent in the MGP, WHR, and WCP regions, and other haplotypes derived from this followed three separate evolutionary routes. Another major haplotype *SSIIIb*-H2 originated directly from *SSIIIb*-H9 and was shared by 57 wild rice accessions along with two Indica cultivars. Wild rice accessions were represented by total eight haplotypes, three of which were present exclusively in wild rice accessions (*SSIIIb*-H9 to *SSIIIb*-H11). Indica cultivars were represented by three different haplotypes, (*SSIIIb*-H1 to *SSIIIb*-H3) where they were nested with the wild rice progenitors. Aus cultivars also showed three haplotypes (*SSIIIb*-H3 to *SSIIIb*-H5), shared by all other cultivar groups including Indica, Japonica, and Aromatic. *SSIIIb*-H3, was shared by wild rice and Indica cultivars, *SSIIIb*-H4 by wild rice and Aromatic, whereas *SSIIIb*-H5 by Aus Japonica. Similar to the Indica and Aus cultivars, Japonica cultivars were distributed in all three evolutionary routes and showed four different haplotypes (*SSIIIb*-H2, *SSIIIb*-H5*-SSIIIb*-H6, *SSIIIb*-H7 and *SSIIIb*-H8, **Figure 8** and **Supplementary Figure S5**). Aromatic rice cultivars including both short grain and Basmati type showed single haplotype (*SSIIIb*-H4), which was shared by two wild and one Aus cultivar. Thus, *SSIIIb* gene showed mixed phylogenic patterns among the cultivated rice.

### *SSIVa* and *SSIVb* Genes for Amylopectin Synthesis

**FIGURE 9:**
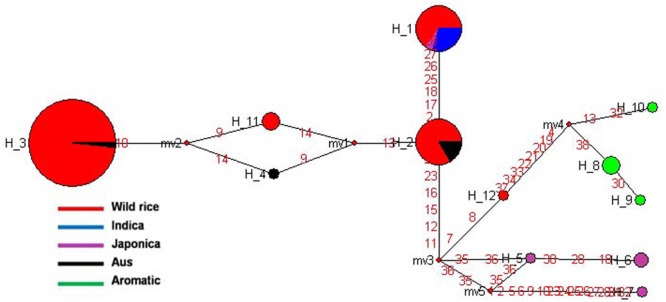
Haplotype networks of *SSIVa* gene, in total 12 haplotypes were formed, and *SSIVa*-H3 was the ancestral haplotype of wild rice along with single Aus FR13A.

*SSIVa* gene located on rice chromosome 1 is one of the least characterized starch synthase genes in plants and is involved in amylopectin biosynthesis. *SSIVa* and *SSIVb* genes are expressed during grain filling in pericarp and endosperm and contribute significantly to grain chalkiness (Hirose and Terao, 2004; Yamakawa et al., 2008). The haplotype network and phylogenetic tree were generated based on 19 SNPs in the *SSIVa* gene from 110 diverse rice genotypes. In total 12 haplotypes were observed, including three major and nine minor haplotypes with ‘Hd’ value of 0.6455 (**Figure 9** and **Supplementary Figure S6**). Here, *SSIVa*-H3 was the most primitive and predominant haplotype shared by 60 wild rice accessions collected from MGP, GPH, and EPH regions and also by a flood tolerant Aus cultivar FR13A. The other two major haplotypes, *SSIVa*-H1 and *SSIVa*-H2 were represented by Indica and Aus cultivars nested in 11 and 15 different wild rice accessions, respectively. *SSIVa*-H1 was also shared by one Japonica cultivar Moroberekan. There were three minor haplotypes each for Japonica and Aromatic cultivars, which were not present in any wild rice accession (*SSIVa*-H5 to *SSIVa*-H7) and (*SSIVa*-H8 to *SSIVa*-H10). Another haplotype *SSIVa*-H4 was represented by a single Aus cultivar. Thus, analysis of *SSIVa* gene also showed that Aus cultivars have the most ancient origin as they carry the most ancestral haplotype common to diverse groups of wild rice ancestors. Once again we notice that like other starch synthase genes cultivated rice has more haplotype diversity than wild rice, suggesting selection for diversified end-usage after domestication.

**FIGURE 10:**
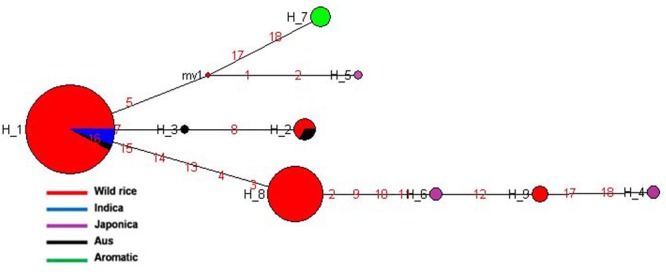
Haplotype networks of *SSIVb* gene, in total nine haplotypes were generated, and *SSIVb*-H1 was the ancestral haplotype combinations of wild rice genotypes and Indica and Aus cultivar groups.

*SSIVb* gene located on rice chromosome 5 plays important role in determining starch granule morphology and in maintaining amyloplast envelope structure. The haplotype network and phylogenetic tree were constructed based on nine SNPs in this gene genotyped in 150 diverse accessions including 130 wild rice accessions and 20 cultivars. There were nine haplotypes, two major and seven minor haplotypes with ‘Hd’ value of 0.5550. *SSIVb*-H1 was the most abundant and ancient haplotype representing 86 wild rice accessions along with five Indica and two Aus cultivars. The 130 wild rice accessions possessed four distinct haplotypes, *SSIVb*-H1 and *SSIVb*-H2 were shared by Aus and Indica cultivars while *SSIVb*-H8 and *SSIVb*-H9 were present exclusively in wild rice accessions (**Figure 10** and **Supplementary Figure S7**). Five Japonica cultivars were grouped into three different haplotypes (*SSIVb*-H4 to *SSIVb*-H6), whereas five Aromatic cultivars showed a separate unique haplotype *SSIVb*-H7. Aus cultivars were also showed three haplotypes(*SSIVb*-H1 to *SSIVb*-H3) two of which were shared with wild rice accessions and Indica where *SSIVb*-H3 was a unique Aus haplotype. Haplotype *SSIVb*-H9 was unique to wild rice accessions collected from MGP region. These results indicate that Japonica rice cultivars originated later from wild rice progenitor and made a separate group. Once again we see three separate phylogenetic routes for the evolution of *SSIVb* gene. There was higher haplotype diversity in Japonica and Aus cultivars as compared to Indica and Aromatic cultivars. These results reveal that evolution of *SSIVb* gene in rice cultivars has a tri-phyletic origin with Aus and Indica cultivars possessing ancient haplotypes.

## Conclusion

The haplotype and phylogenetic analysis of 10 agronomically important key genes for pericarp color, seed size, and starch synthase enzymes suggested a polyphyletic origin of cultivated rice. Here, *Rc, SSSI, SSIIa, SSIIb, SSIIIa*, and *SSIVa* genes showed the biphyletic origin and *GS3, GBSSI, SSIIIb*, and *SSIVb* genes showed the tri-phyletic origin of cultivated rice from their ancestral wild rice. A recent study based on ∼8 million SNPs employing a large data set of 1,083 *O. sativa* and 446 wild rice accessions showed that there were three independent domestications of rice in different parts of Asia (Civan et al., 2015). It has been shown that the Yangtze valley of China is the source of the Japonica rice gene pool, and populations in Indochina and the Brahmaputra valley are the source of the Indica rice gene pool (Civan et al., 2015). Our study showed that the sources of these genes in major rice cultivar groups are genetically diverse wild rice sub-populations. Aus and Indica cultivars have haplotypes common with wild rice populations, whereas haplotypes of Aromatic cultivars including Basmati were closer to Japonica except for *GS3* and *SSIIa*, genes and seem to have evolved more recently. The ancestral haplotypes were shared by wild rice accessions collected from diverse geographical regions including Mid-Gangetic plains, Upper-Gangetic plains, West-Coast plains, and Eastern-Hills of India and three sub-populations of wild rice, namely ‘Pro-Indica,’ ‘Pro-Aus,’ and ‘Mid-Gangetic’ elucidated using a genome wide 48-plex SNP assay (Singh et al., unpublished). Here we showed that Aus cultivars have the most ancient origin as they shared most haplotypes with ancestral wild rice haplotypes, which subsequently may have been diversified into several modern elite varietal groups as confirmed by previous studies (Hazarika, 2006; Kim et al., 2016). These findings will be useful for rice researchers to understand the evolution and domestication of rice and utilization of diverse wild rice germplasm for introgression of novel traits into cultivated rice. Because cultivated rice has less variability as a result of selection during domestication, and the diversity within wild rice genotypes is of immense value for rice improvement especially for impending climate changes.

## Author Contributions

NS conceptualized, designed and conducted the experiments and prepared the manuscript. BS collected maintained and genotyped wild rice. VR, SS, and AS co-supervised the study and NKS conceptualized, supervised the study, edited and finalized the manuscript. All authors read and approved the final manuscript.

## Conflict of Interest Statement

The authors declare that the research was conducted in the absence of any commercial or financial relationships that could be construed as a potential conflict of interest.

## Footnotes

1 http://nksingh.nationalprof.in:8080/iwrdb/index.jsp

2 http://www.medsci.uu.se/digitalAssets/160/c_160695-l_1-k_axiom2.pdf

3 http://www.affymetrix.com

4 http://www.fluxus-engineering.com

5 http://www.ub.es/dnasp

## Supplementary Material

FIGURE S1 Haplotype based phylogenetic tree of *SSSI* gene was constructed using 66 diverse rice genotypes and were separated into 17 different groups. Color coding represents different varietal groups (Cyan-Pro-Indica, Orange-Pro-Aus, Red-Mid-Gangetic, Blue-Indica, Black-Aus, Green-Aromatic, and Magenta-Japonica).

FIGURE S2 Haplotype based phylogenetic tree of *SSIIA* gene was constructed using 175 diverse rice genotypes and were separated into seven distinct groups. Color coding represents different varietal groups (Cyan-Pro-Indica, Orange-Pro-Aus, Red-Mid-Gangetic, Blue-Indica, Black-Aus, Green-Aromatic, and Magenta-Japonica).

FIGURE S3 Haplotype based phylogenetic tree of *SSIIB* gene was constructed using 188 diverse rice genotypes and were separated into six groups. Color coding represents different varietal groups (Cyan-Pro-Indica, Orange-Pro-Aus, Red-Mid-Gangetic, Blue-Indica, Black-Aus, Green-Aromatic, and Magenta-Japonica).

FIGURE S4 Haplotype based phylogenetic tree of *SSIIIA* gene was constructed using 84 diverse rice genotypes and was separated into 18 distinct groups. Color coding represents different varietal groups (Cyan-Pro-Indica, Orange-Pro-Aus, Red-Mid-Gangetic, Blue-Indica, Black-Aus, Green-Aromatic, and Magenta-Japonica).

FIGURE S5 Haplotype based phylogenetic tree of *SSIIIB* gene was constructed using 192 diverse rice genotypes and were separated into 11 major distinct groups. Color coding represents different varietal groups (Cyan-Pro-Indica, Orange-Pro-Aus, Red-Mid-Gangetic, Blue-Indica, Black-Aus, Green-Aromatic, and Magenta-Japonica).

FIGURE S6 Haplotype based phylogenetic tree of *SSIVA* gene was constructed using 110 diverse rice genotypes and were separated into 12 distinct groups. Color coding represents different varietal groups (Cyan-Pro-Indica, Orange-Pro-Aus, Red-Mid-Gangetic, Blue-Indica, Black-Aus, Green-Aromatic, and Magenta-Japonica).

FIGURE S7 Haplotype based phylogenetic tree of *SSIVB* gene was constructed using 150 diverse rice genotypes and were separated into nine major distinct groups. Color coding represents different varietal groups (Cyan-Pro-Indica, Orange-Pro-Aus, Red-Mid-Gangetic, Blue-Indica, Black-Aus, Green-Aromatic, and Magenta-Japonica).
