# Supplementary figures and images for: Evolutionary Insights Based on SNP Haplotypes of Red Pericarp, Grain Size and Starch Synthase Genes in Wild and Cultivated Rice
Source: Front Plant Sci. 2017 Jun 9;8:972. doi: 10.3389/fpls.2017.00972 (PMC5465369; doi:10.3389/fpls.2017.00972)

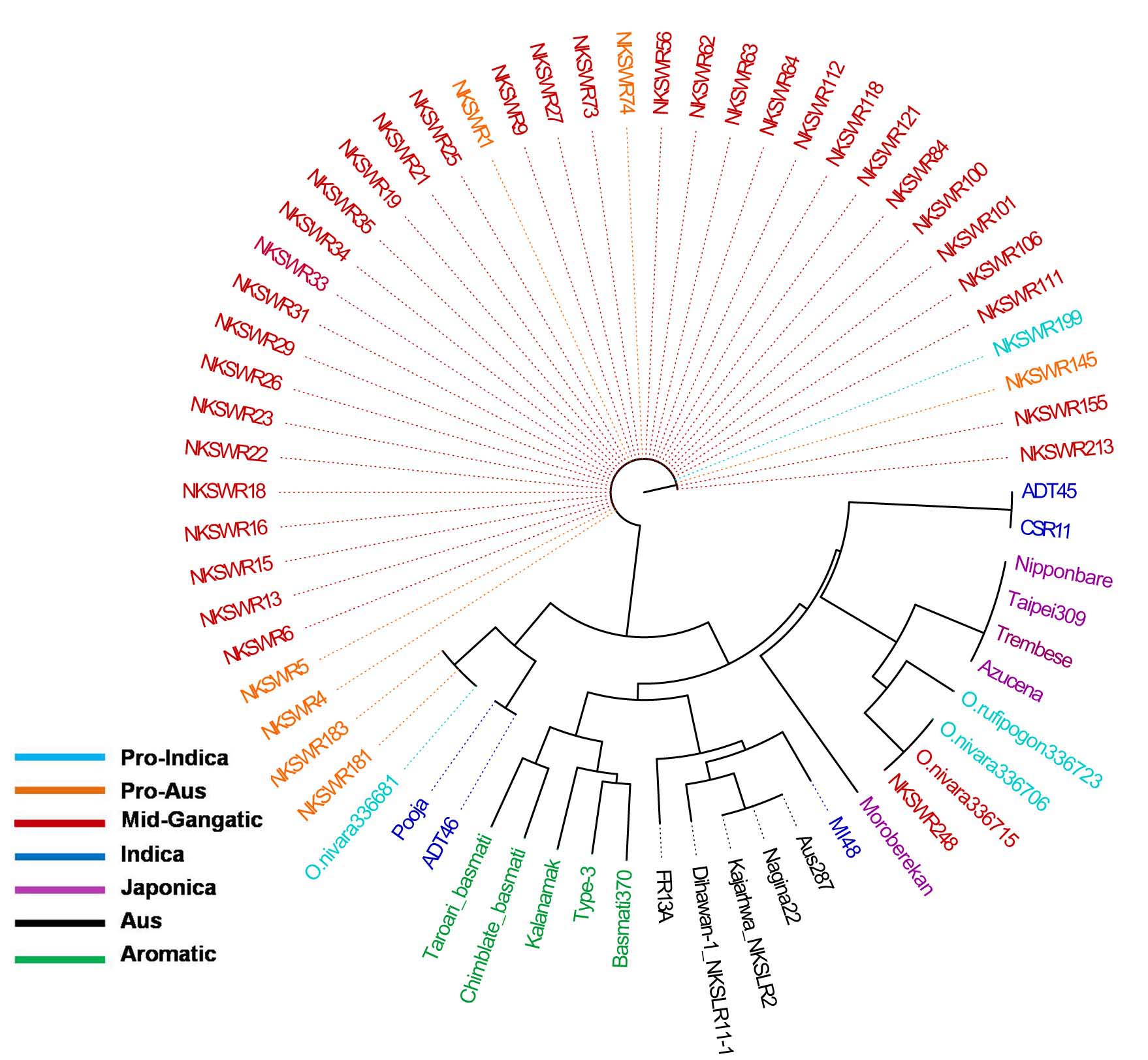

Supplement: FIGURE S1 — Haplotype based phylogenetic tree of SSSI gene was constructed using 66 diverse rice genotypes and were separated into 17 different groups. Color coding represents different varietal groups (Cyan-Pro-Indica, Orange-Pro-Aus, Red-Mid-Gangetic, Blue-Indica, Black-Aus, Green-Aromatic, and Magenta-Japonica). [file Image_1.JPEG]

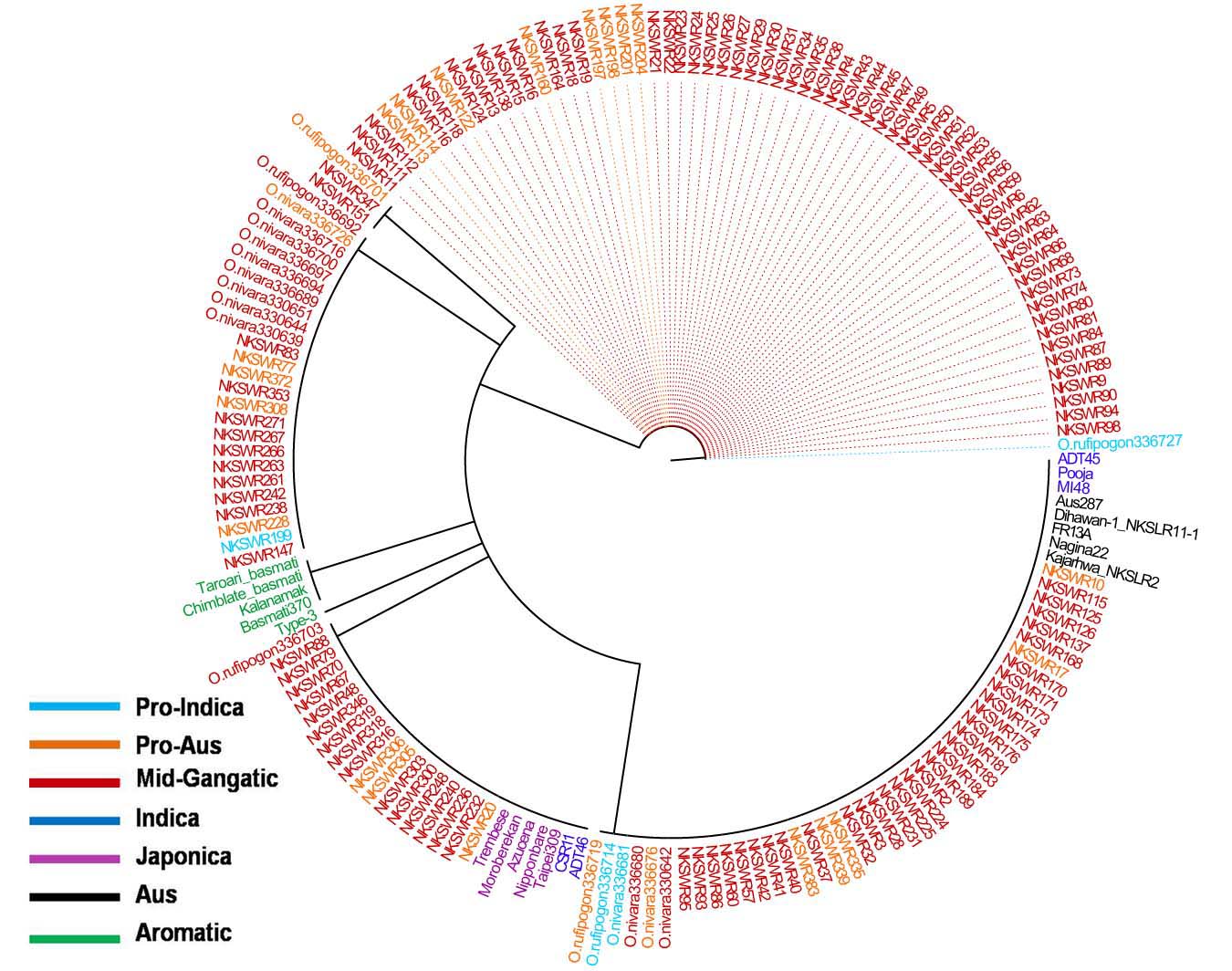

Supplement: FIGURE S2 — Haplotype based phylogenetic tree of SSIIA gene was constructed using 175 diverse rice genotypes and were separated into seven distinct groups. Color coding represents different varietal groups (Cyan-Pro-Indica, Orange-Pro-Aus, Red-Mid-Gangetic, Blue-Indica, Black-Aus, Green-Aromatic, and Magenta-Japonica). [file Image_2.JPEG]

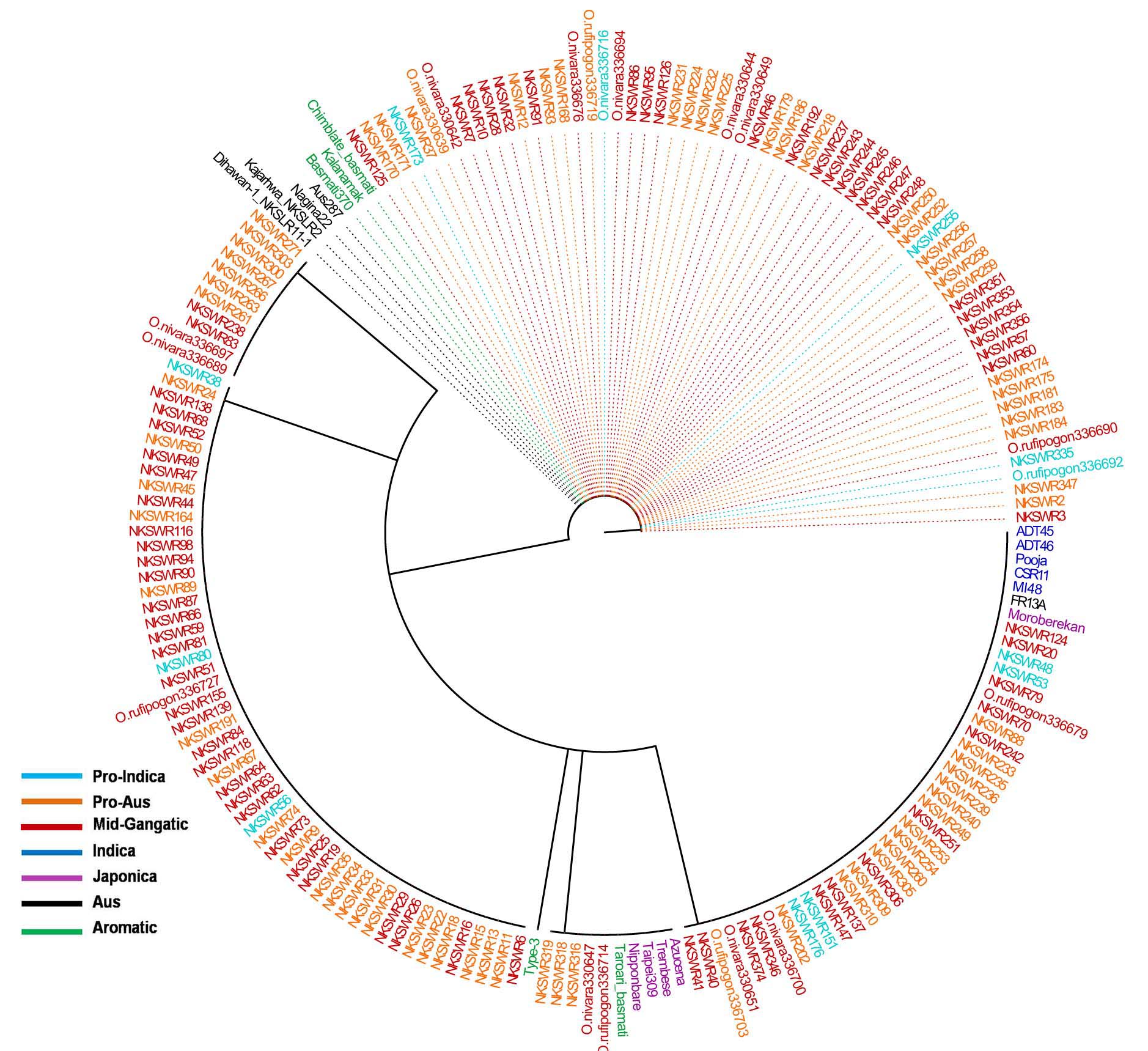

Supplement: FIGURE S3 — Haplotype based phylogenetic tree of SSIIB gene was constructed using 188 diverse rice genotypes and were separated into six groups. Color coding represents different varietal groups (Cyan-Pro-Indica, Orange-Pro-Aus, Red-Mid-Gangetic, Blue-Indica, Black-Aus, Green-Aromatic, and Magenta-Japonica). [file Image_3.JPEG]

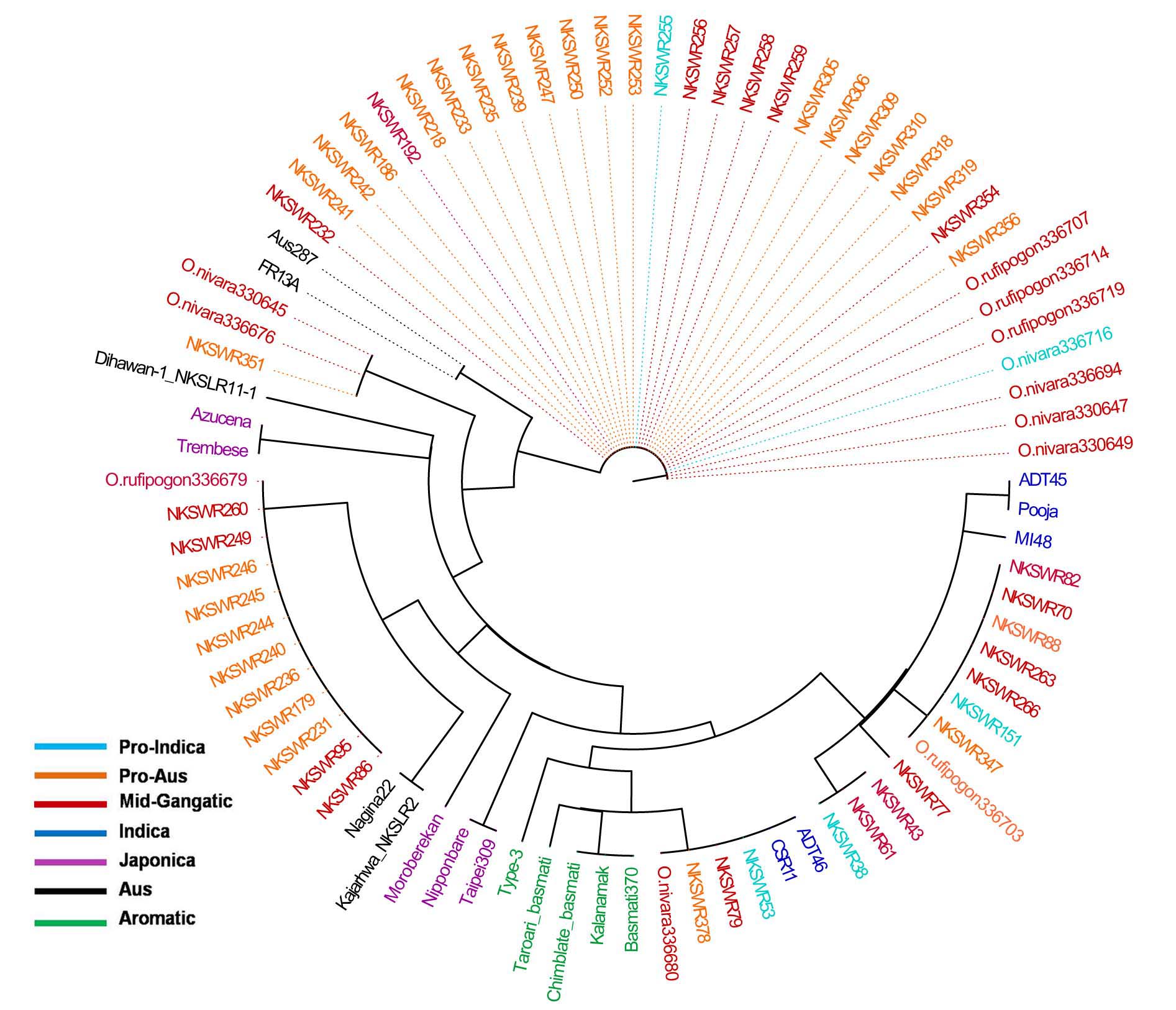

Supplement: FIGURE S4 — Haplotype based phylogenetic tree of SSIIIA gene was constructed using 84 diverse rice genotypes and was separated into 18 distinct groups. Color coding represents different varietal groups (Cyan-Pro-Indica, Orange-Pro-Aus, Red-Mid-Gangetic, Blue-Indica, Black-Aus, Green-Aromatic, and Magenta-Japonica). [file Image_4.JPEG]

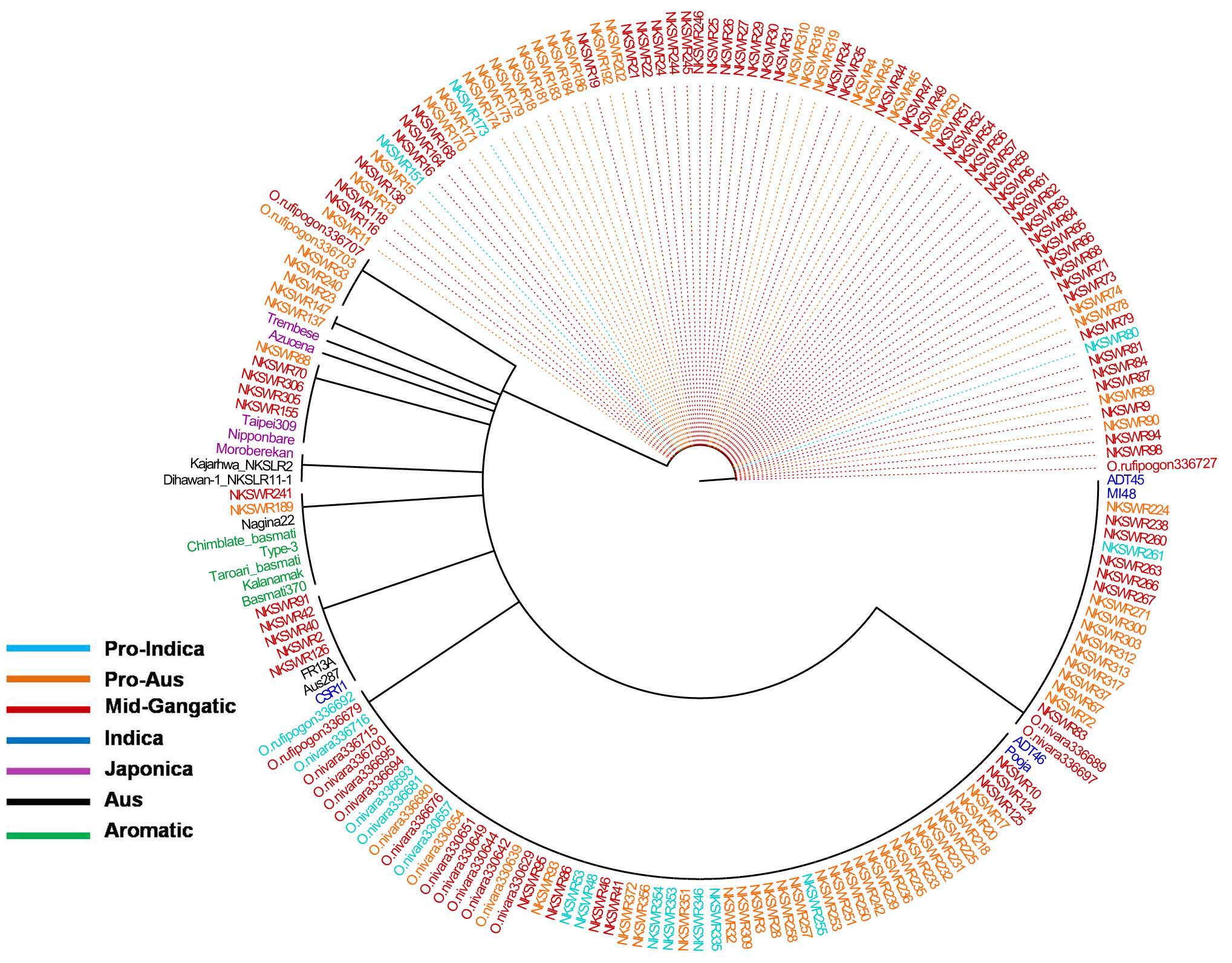

Supplement: FIGURE S5 — Haplotype based phylogenetic tree of SSIIIB gene was constructed using 192 diverse rice genotypes and were separated into 11 major distinct groups. Color coding represents different varietal groups (Cyan-Pro-Indica, Orange-Pro-Aus, Red-Mid-Gangetic, Blue-Indica, Black-Aus, Green-Aromatic, and Magenta-Japonica). [file Image_5.JPEG]

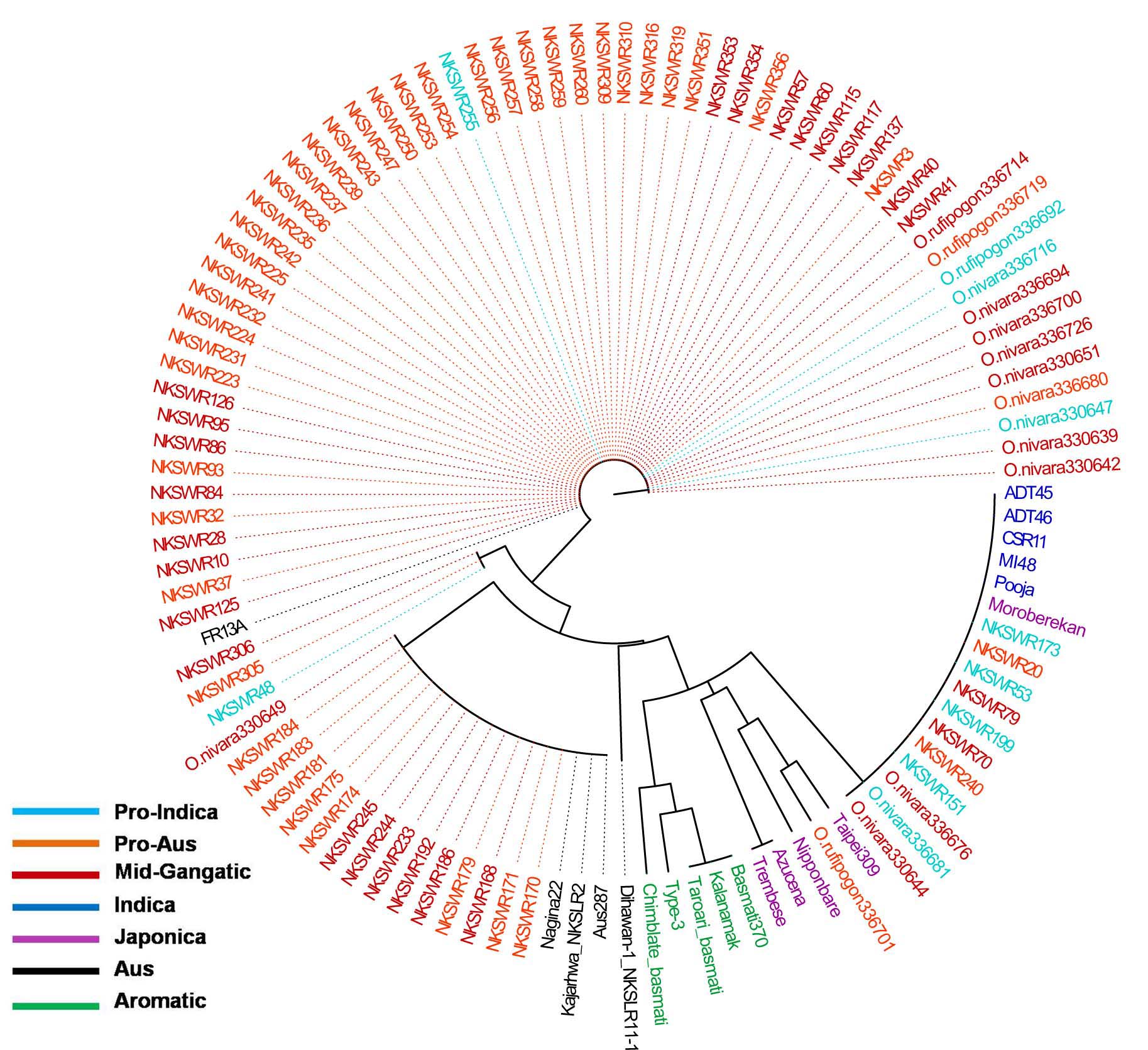

Supplement: FIGURE S6 — Haplotype based phylogenetic tree of SSIVA gene was constructed using 110 diverse rice genotypes and were separated into 12 distinct groups. Color coding represents different varietal groups (Cyan-Pro-Indica, Orange-Pro-Aus, Red-Mid-Gangetic, Blue-Indica, Black-Aus, Green-Aromatic, and Magenta-Japonica). [file Image_6.JPEG]

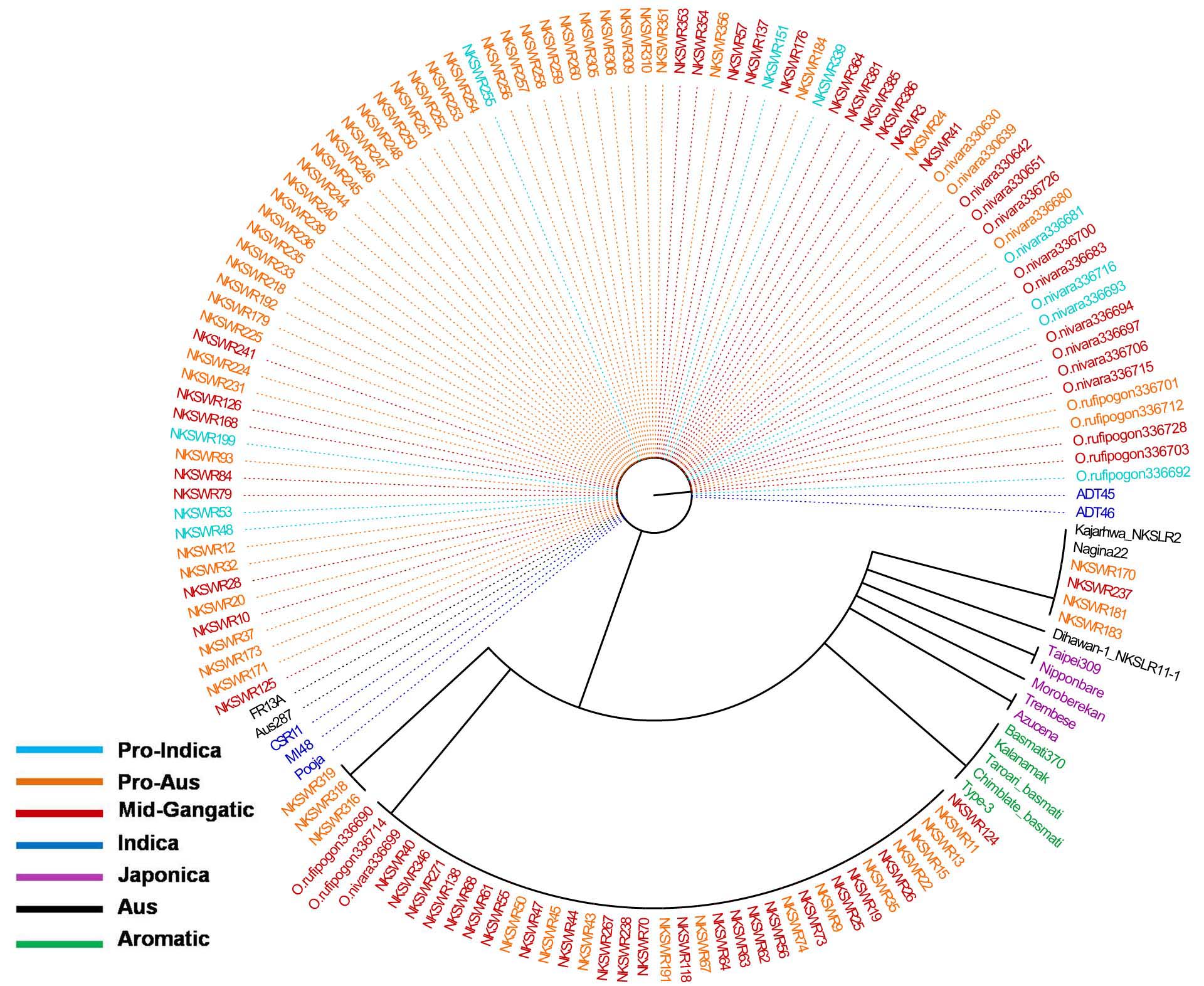

Supplement: FIGURE S7 — Haplotype based phylogenetic tree of SSIVB gene was constructed using 150 diverse rice genotypes and were separated into nine major distinct groups. Color coding represents different varietal groups (Cyan-Pro-Indica, Orange-Pro-Aus, Red-Mid-Gangetic, Blue-Indica, Black-Aus, Green-Aromatic, and Magenta-Japonica). [file Image_7.JPEG]
